# Dual‐center experiences with interventional closure of patent foramen ovale: A medium‐term follow‐up study comparing two patient groups aged under and over 60 years

**DOI:** 10.1002/clc.23548

**Published:** 2021-02-17

**Authors:** Dejan Nachoski, Joerg Schroeder, Mohammad Almalla, Ralf Kubini, Vadim Tchaikovski, Christoph Kosinski, Michael Becker, Ali Aljalloud

**Affiliations:** ^1^ Rhein‐Maas Hospital Department of Cardiology, Nephrology and Internal Intensive Care Würselen Germany; ^2^ RWTH University Hospital Aachen Department of Cardiology, Pulmonology and Internal Itensive Care Aachen Germany; ^3^ Rhein‐Maas Hospital Department of Neurology Würselen Germany; ^4^ RWTH University Hospital Aachen Department of Cardiac Surgery Aachen Germany

**Keywords:** cryptogenic stroke, elder patients, patent foramen ovale, PFO and atrial fibrillation, PFO closure

## Abstract

**Background:**

Current guidelines recommend interventional closure of patent foramen ovale (PFO) in patients with cryptogenic ischemic stroke who are under 60 years of age.

**Hypothesis:**

The hypothesis of this study was to compare follow‐up results of PFO closure in patients over 60 years of age to those of patients under 60 years of age in order to determine whether the procedure is safe and effective for both age groups.

**Methods:**

We included 293 patients who had a cryptogenic ischemic stroke and a PFO confirmed by transesophageal echocardiography (TEE) and who were scheduled for percutaneous closure of the PFO between 2014 and 2019. The device implantation was completed in all patients using an Amplatzer™, Occlutec™, or Cardia Ultrasept PFO occluder.

**Results:**

Follow‐up TEE examinations were performed at intervals of 1, 3, and 6 months after implantation. Patients were followed for a median of 3.6 ± 1.2 years. Recurrent ischemic stroke or transient ischemic attack, cardiac death, arrhythmias, and residual shunt were reported equally in both groups.

**Conclusions:**

Interventional closure of PFO can be as safe and effective in patients over 60 years of age as it is in patients under 60 years of age regardless of the device used. In this older patient group, rigorous discussion and a case‐by‐case decision‐making process including cardiologists and neurologists is warranted to ensure optimal procedure selection.

## 1 INTRODUCTION

Even after in‐depth and extensive investigation, the cause of more than 30% of strokes remains unknown.^1^, ^2^ Patent foramen ovale (PFO) is quite controversially discussed in academic literature as a potential cause of cryptogenic stroke and has been reported to be found in 25% to 40% of the population.^3^

Several early studies asserted that a PFO does not necessarily increase the risk of stroke recurrence.^4^, ^5^, ^6^ Moreover, various researchers using metanalysis failed to find definitive proof that PFO closure reduced the risk of a recurrent stroke.^7^, ^8^, ^9^, ^10^

However, the latest trials conducted with long‐term follow‐up of patients who received PFO closure successfully prove a significant reduction in the recurrence of cryptogenic stroke in patients under 60 years of age.^11^, ^12^, ^13^, ^14^ Following these results, guidelines of the German Societies of Cardiology and Neurology strongly recommended interventional closure for these patients. Furthermore, additional studies report a strong association between the presence of a PFO and cryptogenic stroke.^15^, ^16^, ^17^ Thus, paradoxical embolism through a PFO may be an important cause of otherwise unexplained ischemic cerebral events.^18^, ^19^

Current literature and studies covering the subject matter often fail to include elderly patients.^4^, ^20^, ^21^ Theoretically, patients face increasingly competing sources for embolic stroke with increasing age such as rhythm disturbances like atrial fibrillation or severe cerebrovascular disease.

The role of PFO closure in preventing stroke recurrence in patients older than 60 years remains uncertain particularly after exclusion of these concurrent pathways. PFO is still a potential cause of cryptogenic stroke in both older and younger populations.^22^ Thus, the present dual‐center study was undertaken to retrospectively evaluate the medium‐term clinical results after percutaneous closure using three well‐established occlusion devices in older patients.

## 2 METHODS

### 2.1 Patient population

This study was approved by the local ethical committee. From 2014 to 2019, 293 consecutive patients with a PFO, at least one documented presumed paradoxical thromboembolic event and a scheduled percutaneous closure of the PFO defect were considered for this study. Patients were divided into two groups: patients under 60 years in age (Group A) and patients over 60 years in age (Group B) and signed informed consent forms. All data were analyzed retrospectively. An ischemic stroke was defined as an acute focal neurologic deficit, presumably due to ischemia, that either resulted in clinical symptoms lasting 24 hours or more or was associated with evidence of relevant infarction on magnetic resonance imaging (MRI) or, if MRI could not be performed, by computed tomography (CT) of the brain. All evidence was confirmed by a neurologist where the symptomatic patient first presented. In all cases, a PFO was identified by transesophageal echocardiography (TEE) and other thromboembolic risk factors were excluded, such as, (a) large vessel arteriopathy, (b) an intracardiac thrombus, masses and valve vegetations, (c) intrinsic small vessel disease, (d) cardiac arrhythmias (through 3‐day electrocardiography), and (e) a hypercoagulable state (protein C and S, antithrombin III, antiphospholopid antibodies, and activated protein C resistance).

Post‐intervention bleeding was defined as a decrease of hemoglobin count ≥2 g/dL.

### 2.2 Echocardiography

TEE (iE33, Philips or E90, GE) was performed within 3 ± 2 weeks prior to device implantation (baseline) and at 3‐months and 6‐months follow‐up. Atrial septal aneurysm (ASA) was diagnosed on the basis of a septum primum excursion greater than 10 mm as identified on transesophageal echocardiography. Shunts were defined by the appearance of microbubbles in the left atrium within three cardiac cycles after opacification of the right atrium at rest or during Valsalva maneuver. The degree of shunt was graded into three groups: 1–9 bubbles as ''small,'' up to 30 bubbles as ''moderate,'' and more than 30 bubbles as ''large''. The results of PFO closure were assessed with the use of contrast echocardiography. Furthermore, device position and absence of thrombotic formations were evaluated.

### 2.3 Implantation procedure

All patients received 100 mg of aspirin and 75 mg of clopidogrel daily for at least 1 week prior to intervention. In brief, venous access was gained through the right femoral vein under local anesthesia and device implantation was guided by fluoroscopy and TEE. The type and size of device was chosen by the physician performing the implantation according to (a) the diameter of the defect, (b) presence of an ASA, (c) availability of the device, and (d) appropriate preference. A randomization algorithm was not used.

### 2.4 Post‐implantation treatment

After implantation, patients continued to receive 100 mg of aspirin and 75 mg of clopidogrel daily for 3 months, followed by aspirin monotherapy for up to 6 months. Before hospital discharge, a transthoracic echocardiography was performed to confirm correct device position. Prophylaxis of infectious endocarditis was performed for the first 6 months. Clinical follow‐ups were performed after 3 and 6 months by TEE. Additionally, telephone contact based on a centrally recorded database was conducted on an annual basis. All verification of database recordings and interviews was performed by an experienced nurse or physician. During this interview, the patient or a family member was queried for the occurrence of cardiac events, such as, (a) cardiovascular death, defined as any death with a demonstrable cardiovascular cause or any death that was not clearly attributable to a non‐cardiovascular cause or (b) hospitalization for recurrent neurological or peripheral thromboembolic events. If such an event was identified, the referring general practitioner was contacted for detailed information. No loss of follow‐up occurred. The mean follow‐up period was 3.6 ± 1.2 years, the median monitoring interval was 3.2 years.

### 2.5 Statistics

Continuous data are expressed as mean values ± SD and compared using Student's *t* test or ANOVA as appropriate. Ordinal data were compared using the chi‐square test. A p‐value of <.05 was considered statistically significant. Analysis was conducted using SAS.

## 3 RESULTS

### 3.1 Patient population

The patient population included 293 consecutive symptomatic patients with scheduled percutaneous PFO closure procedures: 192 patients in Group A (mean age 47 ± 6 years) and 101 patients in Group B (mean age 67 ± 4 years). In all patients, Amplatzer™ (Group A, n = 53 patients, Group B, n = 11 patients), Cardia Ultrasept PFO (Group A, n = 95 patients, Group B, n = 77 patients), or Occlutec™ (Group A, n = 44 patients, Group B, n = 13 patients) occluders were implanted. Baseline characteristics are given in Table 1. The demographic and clinical characteristics of the two groups were generally well balanced (Table 1).

**TABLE 1:** Baseline of all included patients

Group A	Amplatzer ™ devices (N = 53)	Cardia Ultrasept PFO devices (N = 95)	Occlutec ™ devices (N = 44)	p
Age, years	46 ± 6	48 ± 7	47 ± 7	0.291
Male sex, n (%)	29 (54)	48 (51)	24 (55)	0.493
Medical history, n (%)				
Diabetes mellitus	3 (5)	4 (4)	5 (11)	0.061
Arterial hypertension	15 (29)	22 (23)	9 (22)	0.243
Smoking	12 (22)	27 (29)	14 (31)	0.149
Hypercholesterolemia	12 (22)	25 (26)	12 (27)	0.477
Coronary artery disease	1 (1)	1 (1)	0 (0)	0.832
Congestive heart failure	1 (1)	0 (0)	0 (0)	0.882
Atrial septal aneurysm, n (%)	19 (36)	33 (35)	14 (33)	0.558
Size of shunt by microbubbles				
Small, n (%)	9 (17)	23 (24)	7 (15)	0.244
Moderate, n (%)	20 (38)	29 (31)	17 (39)	0.342
Large, n (%)	24 (45)	43 (45)	20 (46)	0.638

Group B	Amplatzer ™ devices (N = 11)	Cardia Ultrasept PFO devices (N = 77)	Occlutec ™ devices (N = 13)	p
Age, years	68 ± 3	65 ± 4	67 ± 4	0.332
Male sex, n (%)	5 (48)	42 (55)	7 (54)	0.729
Medical history, n (%)				
Diabetes mellitus	1 (9)	5 (6)	1 (8)	0.438
Arterial hypertension	3 (28)	22 (28)	4 (31)	0.667
Smoking	2 (18)	15 (20)	2 (15)	0.072
Hypercholesterolemia	3 (27)	17 (22)	3 (23)	0.373
Coronary artery disease	1 (9)	3 (4)	1 (8)	0.107
Congestive heart failure	0 (0)	1 (1)	1 (8)	0.078
Atrial septal aneurysm, n (%)	4 (36)	26 (34)	4 (31)	0.592
Size of shunt by microbubbles				
Small, n (%)	2 (18)	15 (19)	2 (15)	0.631
Moderate, n (%)	4 (36)	26 (34)	5 (38)	0.187
Large, n (%)	5 (45)	36 (47)	5 (38)	0.227

### 3.2 Implantation procedure and complication

In the 293 study patients, device implantation failed in none (0) of the patients. Mean procedural time was 29 ± 6 minutes with a mean fluoroscopy time of 2 ± 1 minutes. The number of periprocedural complications was 8 (4%) in Group A and 5 (5%) in Group B with no significant differences between these groups (p = 0.772), see Table 2. One patient in Group A suffered from a severe air embolism, most likely due to a damaged delivery system, with need for hyperbaric oxygen therapy.

**TABLE 2:** Adverse events related to the procedure

Group A	Amplatzer™ devices (N = 53)	Cardia Ultrasept PFO devices (N = 95)	Occlutec™ devices (N = 44)	p
Air embolism, n (%)	0 (0)	0 (0)	1 (2)	0.275
Bleeding, n (%)	1 (2)	2 (2)	0 (0)	0.099
Cardiac perforation, n (%)	0 (0)	0 (0)	0 (0)	n.s
Cardiac thrombosis, n (%)	0 (0)	0 (0)	0 (0)	n.s
Deep vein thrombosis, n (%)	1 (2)	0 (0)	0 (0)	0.172
Device dislocation, n (%)	0 (0)	0 (0)	0 (0)	n.s
Infection/sepsis, n (%)	0 (0)	1 (1)	1 (2)	0.578
Ischemic stroke/TIA, n (%)	1 (2)	0 (0)	0 (0)	0.199
Pericardial effusion, n (%)	0 (0)	0 (0)	0 (0)	n.s
Pulmonary embolism, n (%)	0 (0)	0 (0)	0 (0)	n.s
Total, n (%)	3 (6)	3 (3)	2 (5)	0.536

Group B	Amplatzer™ devices (N = 11)	Cardia Ultrasept PFO devices (N = 77)	Occlutec™ devices (N = 13)	p
Air embolism, n (%)	0 (0)	0 (0)	0 (0)	n.s.
Bleeding, n (%)	0 (0)	1 (2)	0 (0)	0.711
Cardiac perforation, n (%)	0 (0)	0 (0)	0 (0)	n.s
Cardiac thrombosis, n (%)	0 (0)	0 (0)	0 (0)	n.s
Deep vein thrombosis, n (%)	0 (0)	0 (0)	1 (7)	0.037
Device dislocation, n (%)	0 (0)	0 (0)	0 (0)	n.s
Infection/sepsis, n (%)	0 (0)	1 (3)	0 (0)	n.s
Ischemic stroke/TIA, n (%)	0 (0)	0 (0)	0 (0)	n.s
Pericardial effusion, n (%)	0 (0)	0 (0)	0 (0)	n.s
Pulmonary embolism, n (%)	0 (0)	0 (0)	0 (0)	n.s
Total, n (%)	1 (9)	3 (4)	1 (7)	0.074

### 3.3 Endpoints during follow‐up period

Two patients in Group B were lost due to non‐cardiac death (breast cancer, N = 1 and thrombotic thrombocytopenic purpura, N = 1). Follow‐up echocardiography showed a continuous decrease of residual shunting during the 6 months following implantation: full closure was present in 179 patients (93%) in Group A and 96 patients (95%) in Group B with no differences between the groups (p = 0.582), see Table 3. In cases of persistent residual shunt, the patient was instructed to take aspirin (100 mg daily) long‐term. After a mean follow‐up of 3.6 ± 1.2 years, three recurrent thromboembolic events occurred; one event in Group A (1%) and 2 events in Group B (2%) with no significant differences in both groups (p = 0.112). Additionally, we saw new onset of atrial fibrillation in 7 patients in Group A and in 4 patients in Group B. Notably, there were no differences between the groups (p = 0.662).If we look at the final results of both groups we find that there are no significant differences in the event rate in both main groups and subgroups (Table 3).

**TABLE 3:** Events during follow‐up

Group A	Amplatzer™ devices (N = 53)	Cardia Ultrasept PFO devices (N = 95)	Occlutec™ devices (N = 44)	p
Recurrent ischemic stroke/TIA, n (%)	1 (2)	0 (0)	0 (0)	0.832
Cardiac death, n (%)	0 (0)	0 (0)	0 (0)	n.s.
Device related thrombosis, n (%)	0 (0)	0 (0)	0 (0)	n.s.
New onset of atrial fibrillation, n (%)	3 (6)	1 (1)	3 (7)	**0.043**
Residual shunt, n (%)	4 (8)	6 (6)	3 (7)	0.216

Group B	Amplatzer™ devices (N = 11)	Cardia Ultrasept PFO devices (N = 77)	Occlutec™ devices (N = 13)	p
Recurrent ischemic stroke/TIA, n (%)	1 (9)	0 (0)	1 (8)	0.002
Cardiac death, n (%)	0 (0)	0 (0)	0 (0)	n.s.
Device related thrombosis, n (%)	0 (0)	0 (0)	0 (0)	n.s.
New onset of atrial fibrillation, n (%)	2 (18)	1 (1)	1 (8)	**0.032**
Residual shunt, n (%)	1 (9)	3 (4)	1 (8)	0.393

## 4 DISCUSSION

In our dual‐center experience, we highlight that interventional PFO closure could be conducted safely in patients both under and over 60 years of age, significantly reducing the recurrence of stroke in both populations equally. The generally accepted recommendation that patients over 60 years old should be denied treatment in the form of an interventional PFO closure should be reconsidered, especially when taking into account the aging demographics of the world's population.

The results of our study underscore that age alone should not be a primary exclusionary factor when deciding whether an interventional treatment is suitable for a patient with PFO. Physicians are instead advised to take further criteria, such as, medical history and general health, into consideration on a case‐by‐case basis when determining the best course of action for each individual. In all device types, PFO occlusion was found to be safe and effective in patients over 60 years of age compared with patients under 60.

Our study has a few limitations, specifically that it is a small non‐randomized observational study. Furthermore, follow‐ups were performed by TEE examinations after 3 and 6 months and through telephone contact with the patient and the practitioner regarding cardiovascular or neurological events. However, there may be a recall bias and an opportunity to miss or to over‐interpret clinical events.

## 5 CONCLUSION

In cases of patent foramen ovale following cryptogenic ischemic stroke, patients over 60 years of age could also be considered suitable candidates to receive interventional closure of the PFO in order to substantially reduce the risk of a recurrent stroke, and age alone should not be considered a limiting factor. Based on the results of this study, interventional closure of patent foramen ovale could be as safe and effective in patients over 60 years of age as it is in patients under 60 years of age. In order to determine the best treatment options, the patient's cardiologist and neurologist are tasked with deciding on the optimal course of action based on the individual patient's medical history and current physical condition. Since it is linked to the increasing life expectancy and improved quality of life of the population, this topic will occupy us doctors more in the future. For this purpose, in order to make a strong definitive statement, we need multi‐center randomized large studies.

## CONFLICT OF INTEREST

The authors declare no potential conflict of interest.

## DATA AVAILABILITY

All data are available at the Rhein‐Maas Hospital, covered by Prof. Michael Becker.
